# Antimicrobial Use and Resistance in Animals from a One Health Perspective

**DOI:** 10.3390/vetsci10050319

**Published:** 2023-04-28

**Authors:** Mohamed Rhouma, Marie Archambault, Patrick Butaye

**Affiliations:** 1 Department of Pathology and Microbiology, Faculty of Veterinary Medicine, Université de Montréal, 3200 Rue Sicotte, Saint-Hyacinthe, QC J2S 2M2, Canada; 2 Groupe de Recherche et d’Enseignement en Salubrité Alimentaire (GRESA), Faculty of Veterinary Medicine, Université de Montréal, Saint-Hyacinthe, QC J2S 2M2, Canada; 3 Swine and Poultry Infectious Diseases Research Center, Faculty of Veterinary Medicine, Université de Montréal, Saint-Hyacinthe, QC J2S 2M2, Canada; 4 Department of Pathobiology, Pharmacology and Zoological Medicine, Faculty of Veterinary Medicine, Ghent University, Salisburylaan 133, B9820 Merelbeke, Belgium

Among the many global health issues, antimicrobial resistance (AMR) is one that exemplifies the One Health approach, defined as a joint effort in which multiple disciplines collaborate to provide solutions for human, animal, and environmental health [1]. Indeed, AMR is predominantly the result of the irresponsible and excessive use of antimicrobials in various sectors, such as human/veterinary medicine and agriculture (e.g., plant health). Accordingly, solutions must be contributed by all relevant stakeholders to address this global issue. The real dangers posed by the rapid loss of antimicrobial effectiveness, which has been precipitated by the selection and spread of AMR bacteria, have prompted policy makers to recognize this threat to health care systems and the economy while considering it as a budgetary and regulatory priority [2]. Moreover, the COVID-19 pandemic has generated powerful incentives and momentum with respect to raising awareness of the importance of having effective drugs to treat microbial infections and the need to ensure their efficacy over time [3]. As the number of brand-new antimicrobials available on the market has drastically decreased since the 1990s [4], it is therefore crucial to implement all possible strategies, in both human and veterinary medicine, to minimize the selection of AMR bacteria while preserving the efficacy of the existing drugs and ensuring their optimal longevity. Notably, the protection of the current antimicrobial arsenal is of particular concern in veterinary medicine, where the possibility of acquiring new antimicrobials is extremely limited and drug repurposing has been scarcely explored (unlike the trend in human medicine) [4].

Hence, the theme of this Special Issue is very topical and of particular interest to the various stakeholders involved in the management of AMR in animals and at the human–animal–environment interface. Indeed, several studies have shown that the misuse and excessive use of antimicrobials in the human health care and livestock industries are the main drivers of AMR [5,6,7]. How livestock contributes to the spread of AMR in humans continues to be studied. The most recent studies do not quite indicate that there is a large impact of AMR in animal bacteria on AMR in human bacteria, with the exception of zoonotic bacteria [7,8,9]. However, it was recently shown that the effect of antimicrobial use (AMU) on AMR have a bidirectional influences on AMR in human and animal bacteria on a global scale [10]. These recent findings once again underscore the critical need to accelerate the implementation of the One Health approach to address the global AMR crisis.

Among the possible solutions, research on alternatives to antibiotics for use in animals seems necessary to contain the threat of AMR. For instance, it has been reported that Romanian propolis ethanolic extracts present significant in vitro antibacterial activity against clinical strains of *Staphylococcus aureus* isolated from dog-derived superficial dermatitis samples and could, therefore, constitute a promising therapeutic option against this potential skin infection [11]. Likewise, silver nanoparticles (AgNPs) present in vitro antibacterial activity against some bacterial pathogens (e.g., *Staphylococcus pseudintermedius*, *Staphylococcus aureus*, *Escherichia coli*) and low toxicity to mammalian cells, suggesting that AgNPs could be used as an alternative to antibiotics for the treatment of bacterial skin infections, including infected wounds in animals [12].

The monitoring of AMR in animals and the characterization of the AMR patterns of certain indicator bacterial strains are of crucial importance for the development of policies that will ensure the responsible use of antimicrobials in animals. The development of standardized AMR testing methods and harmonized interpretive criteria in veterinary medicine are necessary to facilitate scientifically based stewardship and justified AMU in animals [13]. It has also been shown that the susceptibility testing of bacteria, which are not routinely investigated for AMR profiling, should sometimes be incorporated in AMR surveillance systems alongside the consideration of the epidemiological and microbiological data specific to certain countries [14] or to certain animal production sectors (e.g., the fish-farming sector) [15].

Moreover, AMR in zoonotic bacteria (e.g., *Salmonella*) remains problematic in some countries, with high rates of bacterial resistance to first- and second-line antimicrobials being reported, thus urging various stakeholders in animal production to implement effective policies to limit the spread of AMR [16].

Drug repurposing and repositioning, which consist of identifying new therapeutic uses for existing molecules, have been scarcely investigated in veterinary medicine. In this regard, it has been shown that the anti-inflammatory properties of doxycycline may be useful for the long-term treatment of severe bronchiectasis in dogs [17].

Finally, in an overview of AMU in food-producing animals and of the current state of knowledge regarding the role of farm animals in the spread of AMR in humans, actions taken in the livestock industry were presented, from a sustainable animal production perspective, in order to limit the spread of AMR bacteria and preserve the effectiveness of antimicrobials [18].

Notably, the Guest Editors of this Special Issue have remarked that despite the growing interest in veterinary antimicrobial stewardship (AMS), the optimization of the therapeutic use of antimicrobials (with respect to dose, administration intervals, and treatment durations), which constitutes the key component of this concept, has not received the attention it deserves in animals compared to that seen in human medicine, and this aspect must be improved in future studies.
